# Editorial: Exploring autoimmune diseases and endocrine crosstalk

**DOI:** 10.3389/fimmu.2025.1709567

**Published:** 2025-10-09

**Authors:** Anette S. B. Wolff, Alessandro Antonelli

**Affiliations:** ^1^ Department of Medicine, Haukeland University Hospital, Bergen, Norway; ^2^ Department of Biomedicine, University of Bergen, Bergen, Norway; ^3^ Department of Surgery, Medical and Molecular Pathology and Critical Area, University of Pisa, Pisa, Italy

**Keywords:** endocrine-immune interaction, complexity, autoimmune, immune-metabolic axis, biomarkers

Autoimmune endocrine diseases are increasingly recognized as complex, multifactorial conditions that arise from a breakdown in immune tolerance caused by genetic susceptibility and uncharacterized environmental triggers, and result in targeted destruction of hormone-producing organs. These disorders, which include Hashimoto’s thyroiditis, thyroid eye disease, autoimmune polyendocrine syndromes, and endocrine involvement in systemic autoimmune diseases, are not only clinically diverse but also mechanistically intricate. The crosstalk between the immune and endocrine systems plays a pivotal role in both maintaining physiological homeostasis and driving disease when dysregulated. This Research Topic brings together nine original and review articles that collectively explore the immunological, genetic, metabolic, and environmental dimensions of autoimmune endocrine diseases.

A central theme across several contributions was thyroid autoimmunity, which remains one of the most prevalent and studied forms of organ-specific autoimmunity. One novel study explored the role of tryptophan metabolism in the pathogenesis of Hashimoto’s thyroiditis (HT), demonstrating that HT patients exhibit significantly reduced serum tryptophan levels. Supplementation with tryptophan alleviated thyroid inflammation and tissue damage, while pharmacological inhibition of tryptophan metabolism exacerbated disease severity. Mechanistically, tryptophan was shown to modulate T cell subset distribution and influence the PI3K-Akt signaling pathway, suggesting that metabolic pathways may serve as viable therapeutic targets in HT (Zhang et al.). This underscores the importance of metabolic-immune interactions in endocrine autoimmunity.

Oxidative stress has long been suspected as a contributing factor in thyroid eye disease (TED), but its molecular underpinnings have remained elusive. A bioinformatics-driven study identified 53 oxidative stress-related differentially expressed genes (OS-DEGs) in TED, with FOS, MCL1, and ANGPTL7 emerging as key candidates. These genes were validated through immunohistochemistry in orbital tissues, confirming their upregulation in TED patients. The findings implicate mitochondrial dysfunction and reactive oxygen species (ROS) metabolism in TED pathogenesis and propose these genes as potential biomarkers for early diagnosis and risk stratification (Hai et al.). In a complementary study, the therapeutic potential of Linsitinib, a small-molecule inhibitor of IGF-1R phosphorylation, was evaluated in cell lines expressing IGF-1R and TSH-R, both of which are implicated in TED. Linsitinib effectively inhibited cell proliferation and induced apoptosis (Luffy et al.). These results suggest that dual targeting of IGF-1R and TSH-R signaling may offer a promising strategy for managing TED.

Mavridou and Pearce highlighted that the genetic basis of organ-specific autoimmunity is still not fully understood. The authors explained how polymorphisms in genes encoding endocrine-specific antigens, such as INS, TSHR, TPO, CYP21A2, and PIT-1, can impair central tolerance through mechanisms including altered thymic expression, alternative splicing, and post-translational modifications (Mavridou and Pearce). These insights are crucial for understanding why certain endocrine tissues are preferentially affected and may inform future strategies for disease prediction and prevention.

The clinical complexity of syndromes in which several endocrine conditions appear in concert was illustrated in a case report of autoimmune polyendocrine syndrome type 2 (APS-2), which involves Addison’s disease, Hashimoto’s thyroiditis, and type 1 diabetes mellitus (Yao et al.). The patient presented with an adrenal crisis after a nine-year delay in diagnosis, highlighting the challenges of recognizing asynchronous multi-glandular involvement. The case also raised concerns about the potential impact of exogenous substances on immune balance and hormone metabolism. This underscores the need for vigilant, multidisciplinary monitoring of patients with known autoimmune conditions.

Pregnancy introduces unique immunological and hormonal dynamics, and hypothyroidism during gestation poses significant risks to both maternal and fetal health. A metagenomic and proteomic study investigated the gut microbiota and immune profiles of pregnant women with hypothyroidism. The results revealed reduced microbial diversity and enrichment of *Phocaeicola vulgatus* and *Bacteroides fragilis*, alongside downregulation of DGKK and S10A8 proteins (Zhang et al.). These changes were found to be associated with a shift in the Th1/Th2 balance, suggesting that microbial and immune alterations may contribute to disease onset. This study adds to the growing body of evidence linking gut microbiota to endocrine and immune health.

Systemic autoimmune diseases may also manifest symptoms in endocrine organs. In primary Sjögren’s syndrome (pSS), thyroid involvement is a common but often under-recognized complication. A retrospective study of 202 pSS patients resulted in the development of a predictive nomogram that incorporates clinical and serological variables such as high-sensitivity CRP, Ro52, AST, dryness symptoms, anxiety, and hyperuricemia. This tool can aid clinicians in identifying pSS patients at risk for thyroid dysfunction, enabling earlier intervention and more personalized care (Yang et al.). The systemic nature of autoimmune diseases was further exemplified in a study examining the risk of type 2 diabetes mellitus (T2D) in patients with rheumatoid arthritis (RA). Among 488 RA patients, those with comorbid T2D exhibited a longer disease duration, a higher BMI, and an increased prevalence of hypertension and a family history of diabetes. Immunologically, these patients had reduced Th2 and Treg cell populations, leading to elevated Th1/Th2 and Th17/Treg ratios. A multivariate analysis identified immune cell imbalance, systemic inflammation, and metabolic factors as key contributors to T2D risk in RA patients, highlighting the importance of immune-metabolic crosstalk in the development of comorbidities (Pei et al.). Another article in this Research Topic focused on SLE, with its findings on dyslipidemia and immune activation in SLE highly relevant to endocrine autoimmunity (Xuan et al.). Another article in this Research Topic focused on SLE, which may share immunometabolic pathways with endocrine autoimmune disorders; hormones influence lipid metabolism, patients with SLE often have secondary autoimmune endocrine disorders such as thyroid disease or type 1 diabetes and glucocorticoids are frequently used as therapy for systemic autoimmune disorders. Therefore, biomarkers of these disorders may be the same or influence each other. These studies underscore the importance of immune-metabolic crosstalk and shared inflammatory pathways, along with the need for the integrated management of autoimmune diseases that span both systemic and endocrine domains.

Together, the articles in this Research Topic provide a comprehensive and nuanced view of different areas of autoimmune endocrine diseases. They emphasize the importance of integrating immunological, genetic, metabolic, and clinical data to unravel the complexities of immune-endocrine interactions. From molecular mechanisms and biomarker discovery to predictive modeling and therapeutic innovation, these studies pave the way for improved diagnostics, personalized risk assessment, and targeted treatments.

As the prevalence and complexity of autoimmune endocrine diseases continue to rise, interdisciplinary collaboration will be essential to fostering the development of precision medicine approaches. We hope that this Research Topic will inspire further research and clinical innovation aimed at improving the lives of patients affected by these challenging disorders.

